# Generalized anxiety disorder screening using gad-7 among in-school adolescents of anambra state, nigeria: a comparative study between urban and rural areas

**DOI:** 10.1186/s13034-023-00637-4

**Published:** 2023-07-19

**Authors:** Ahoma Victor Mbanuzuru, Rechard Uwakwe, Chinekwu Sochukwu Anyaoku, Anastacia Okwudili Ojimba, Mary Chinyere Mbanuzuru, Chijioke Amara Ezenyeaku, Darlington Chukwudinma Obi, Christiana Nkiru Okafor, Uchenna Prosper Okonkwo

**Affiliations:** 1 Department of Community Medicine & Primary Health Care, Nnamdi Azikiwe University, Awka, Anambra State Nigeria; 2 Department of Psychiatry, Nnamdi Azikiwe University, Awka, Anambra State Nigeria; 3 Department of Family Medicine, Nnamdi Azikiwe University Teaching Hospital, Awka, Anambra State Nigeria; 4 Center for Communicable Disease Control and Research, Federal Medical Centre Asaba, Asaba, Delta State Nigeria; 5 Animal Production Department, Ministry of Agriculture, Awka, Anambra State Nigeria; 6 Department of Community Medicine, Nnamdi Azikiwe University, Awka, Anambra State Nigeria; 7 Department of Community Medicine and Primary Health Care, Faculty of Medicine, College of Health Sciences, Nnamdi Azikiwe University, Awka, Anambra State Nigeria; 8 Department of Nursing, Faculty of Health Sciences and Technology, Nnamdi Azikiwe University, Awka, Anambra State Nigeria; 9 Department of Medical Rehabilitation, Faculty of Health Sciences and Technology, Nnamdi Azikiwe University, Awka, Anambra State Nigeria

**Keywords:** Prevalence, Anxiety disorder, Adolescents, Urban and rural areas, School, Anambra State

## Abstract

**Background:**

Previous studies have reported that general anxiety disorder manifestations differ in diverse settings.

**Objective:**

To determine and compare the prevalence of probable anxiety disorders among in-school adolescents in urban and rural areas of Anambra State.

**Methods:**

A total of 1187 in-school adolescents were recruited using a multi-stage sampling technique. The study instrument was an interviewer-administered pretested questionnaire adopted from General Anxiety Disorder (**GAD-7**). Data were analyzed with the IBM Statistical Package for the Social Sciences (SPSS) version 22.

**Result:**

One hundred and twenty of the participants out of the 1187 (10.1%) were found to have probable generalized anxiety disorders using GAD-7 as screening tool. The prevalence of symptoms of anxiety disorder revealed that urban participants had a higher prevalence compared to their rural counterparts (11% vs. 8.8%), while females had a higher prevalence compared to the males in the ratio of 3:2 (or 12% vs. 8%). The prevalence of symptoms of anxiety disorders among females was higher than that of males even when compared based on a rural and urban settings. When all other variables are held constant, urban participants were found to have a 50% higher chance of being identified with anxiety disorders compared to their rural participants (OR = 1.500, C.I.:1.002–2.246, p = 0.049).

**Conclusion:**

The prevalence of probable anxiety disorders was found in 10% of the participants. The females have a higher propensity to exhibit symptoms of anxiety disorders than the males. Anxiety status affects how adolescents view their general health. The study started from the date of approval by the West African College of Physicians on the 21 February 2017, but Ethical Clearance from NAUTHEC was given on the 19th December 2016.

## Introduction

Anxiety disorders are generally common, especially in children and adolescents and may be associated with significant suffering, disruption of normal psychosocial and academic development, disruption of family functions, and increased utilization of medical services [1]. Anxiety is an unpleasant emotional state of mental uneasiness, nervousness, apprehension and obsession or concern about some uncertain events. People usually experience anxiety about events they cannot control or predict, or about events that seem threatening or dangerous [2]. When anxiety becomes persistent, seemingly uncontrollable, and overwhelming, excessive leading to irrational dread of everyday situation, it is then known as anxiety disorder [3]. Anxiety disorders include the following: generalized anxiety disorder (GAD), panic disorder, agoraphobia, social anxiety disorder, selective mutism, separation anxiety and specific phobias. Sometimes some other mental disorders such as obsessive-compulsive disorder (OCD) and post-traumatic stress disorder (PTSD) co-occur with anxiety disorders. Childhood and adolescence are the core risk phase for the development of anxiety symptoms and syndromes, ranging from transient mild symptoms to full-blown anxiety disorders [4].

Urban and rural areas do not have equal distribution of social amenities, access to health services and other environmental factors. It is possible that these two settings may have different propensities for the manifestation of anxiety disorders among adolescents. Schools in the urban areas are expected to have more facilities compared to their rural counter-parts. On the other hand, students in the rural areas are exposed to more manual labours compared to their urban peers. Socio-economic status has been described as one of the determinants of mental health [5]. Some studies have reported no significant difference, while others indicated that adolescents living in rural areas appear to have a heightened risk for developing a mental health problem compared to their urban counterparts [5, 6]. The difference between rural and urban communities were noted as a source of divergence in mental health services, with increased stigma in rural communities where anonymity is difficult to be maintained [7]. In a meta-analysis by Peen et al. in 2010, urban-rural differences exist in psychiatric disorders [8]. In Nigeria, Ilesanmi et al. studied the psychological effects of rural versus urban environment on adolescent behaviour following pubertal changes, and found that environment has influence on the adolescents, though the difference was not statistically significant [9]. This work will shed more lights on possible differences between the rural and urban areas in the symptoms of generalized anxiety disorder.

The prevalence and the epidemiology of different anxiety disorders in adolescence were noted to be different from that in adulthood [10]. Studies on prevalence of adolescent anxiety disorder in different parts of the world present very different and diverse reports. The report by the Surgeon General of United States of America (USA) on mental health, revealed that the combined prevalence of the group of disorders known as anxiety disorders is higher than that of virtually all other mental disorders of childhood and adolescence, with one-year prevalence of 13% for those aged between 9 and 17 years [7]. A co-morbidity survey in USA to assess Diagnostic and Statistical Manual-4th edition (DSM-IV) mental disorders among adolescents aged 13 to 18 years using modified version of the fully structured World Health Organization Composite International Diagnostic Interview [11]. revealed the following prevalence: anxiety disorders (31.9%), behaviour disorders (19.1%), mood disorders (14.3%) and substance use disorders (11.4%) [11]. Using Structured Child and Adolescent Psychiatric Assessment and its upward extension, the ‘great smoky mountain study’ in the South-Eastern USA, showed that 20% of subjects met the criteria for an anxiety disorder by early adulthood [12]. A systematic review and meta-regression on prevalence of anxiety disorders among children, adolescents and adults revealed that the global prevalence of anxiety disorders ranged between 0.9% and 28.3%. After adjustment for methodological differences, the global current prevalence of anxiety disorders was given as 7.3% (95% C.I. 4.8 − 10.9%) and ranged from 5.3% (95% C.I. 3.5 − 8.1%) in African cultures to 10.4% (95% C.I. 7.0 − 15.5%) in Euro/Anglo cultures [13]. Similar meta-analyses yielded global prevalence for anxiety in children and adolescents as 6.5% and range 3–22% respectively [14, 15]. Another study for western countries gave life time prevalence of anxiety to range from 13.6 to 28.8% [16] A study in Uganda recorded a prevalence of 26.6% in children and adolescents, using the MINI International Neuropsychiatric Interview for children and adolescents (MINI KID);[17]while 12.9% was reported for Kenyan students using Multidimensional Anxiety Scale for Children (MASC) [18]. From South-West Nigeria, the 12-month prevalence of DSM-IV anxiety disorders among school adolescents was 15% [19].

The dearth of studies on the prevalence of anxiety disorders in in-school adolescents has created a gap that this current study wants to fill. Hence, this study intends to answer the following question, what is the prevalence of anxiety disorders among in-school adolescents in urban and rural areas of Anambra State? It is hereby hypothesized that the level of anxiety disorders among in-school adolescents in urban and rural areas of Anambra State will not be statistically significantly.

## Materials and methods

This cross-sectional comparative study was done among secondary school adolescents (urban and rural), aged 10 to 19 years in government owned schools within the chosen LGAs in Anambra State. The State has its capital and seat of government at Awka, with twenty-one LGAs and three senatorial zones namely Anambra South, Anambra Central and Anambra North [20]. The name Anambra was derived from the **Omambala River** (Anambra River) which is a tributary of the River Niger. The boundaries of Anambra State are formed by Delta State to the west, Imo State and Rivers State to the south, Enugu State to the east and Kogi State to the north. The major ethnic group of the State is Igbo (98% of the population), and a small population of Igala (2% of the population) living mainly in the north-western part of the State [21]. Other ethnic groups in Nigeria (from different tribes and culture) and even non-Nigerians, also live in the State as civil servants and for businesses. Christianity is the predominant religion, while others are Traditional African religion and Islam. Anambra State has 642 secondary schools, 257 of which are owned by the government (public secondary schools). The State has literacy rate of 70% [21].

A minimum sample size of 942 was calculated to determine a difference in urban and rural in-school adolescents with anxiety disorders at 80% power and 95% confidence level. A total of 1187 in-school adolescents were studied at the end, adding more to the power of the study. Those eligible for inclusion in the study were adolescents aged 10–19 years in secondary schools in Anambra State at the time of the study. However, those who met the inclusion criteria but refused to grant assent, or whose informed permission could not be obtained were excluded from the study. Also, those who were aged 18–19 years who refused to give consent, or who out of obvious cases of ill health were not included.

A multi-stage sampling technique was used in this study. At first stage, an urban and rural LGAs were selected from each senatorial zone by simple random sampling method (employing simple balloting). From Anambra North senatorial zone, Onitsha South LGA and Anambra-West LGA were the urban and rural LGAs respectively, while Awka South and Awka North were used as urban and rural LGAs respectively from Anambra Central senatorial zone. In the Anambra South senatorial zone, Nnewi North and Orumba North were selected as the urban and rural LGAs respectively. Proportionate allocation was employed in the second stage to select eighteen schools, taking cognizance of the varied number of schools in each LGA within the 3 senatorial zones. The third stage selection of classes, where both junior and senior secondary arms of the schools were considered for better spread. At the fourth stage, the participants were selected from the chosen class by employing systematic random sampling. From the school records, the population of the urban schools ranged from 600 to 2000, while that of the rural schools ranged from 300 to 600. The implication was that the number of participants needed from the rural schools ranged from 45 to 60, while that of the urban schools ranged from 60 to 90 participants. This gave the average expected number of participants per school as 50 for rural, and 70 for urban schools. This translated to average of 8 participants per class in the rural areas, and 12 participants per class in the urban areas. The sampling interval, k was calculated as appropriate, while the first participant with k was chosen using simple random sampling.

### Study instrument

The study instrument was an interviewer-administered pretested questionnaire adopted from General Anxiety Disorder (**GAD-7**), which is a brief self-report scale but with good reliability and validity [22]. The total score of ≥ 10 is used as screening cut-off point for further evaluation [22]. Clinical evaluation was not included in this study to make definitive diagnosis of anxiety. GAD-7 of 10 and above has been supported for screening/diagnosis of Generalized anxiety. The scores obtained in GAD-7 ranges between 0 and 21, with scores of 5, 10, and 15 representing the cut-points for mild, moderate and severe anxiety respectively [22]. Increasing scores on the scale of GAD-7 were found to be strongly associated with multiple domains of functional impairment [22].

### Ethical consideration

Prior to collection of data, permission was obtained from the Government of Anambra State Post Primary Schools Service Commission-Headquarters Awka, Principals of the respective schools and the Ethics Committee of Nnamdi Azikiwe University Teaching Hospital, Nnewi (NAUTHEC). Informed consent was obtained from each participant aged more than 18 years and assent from those less than 18 years, freely and without coercion after thorough explanation of the study. Informed permission was also sought from parents/guardians through the various school authorities. The participants were then guided to answer the questions in the study instrument to ensure quality data. The work started from the date of approval by the West African College of Physicians in on the 21 February 2017, but Ethical Clearance from NAUTHEC was given on the 19th December 2016. The data collation lasted for three months, and data analysis, results presentation, and discussion of the outcomes lasted for two months. Overall, it took five months to complete the study.

### Data management

Data analysis was done using the IBM Statistical Package for the Social Sciences (SPSS) version 26 [23]. Anxiety was used as the dependent (outcome) variables in this study. The independent variables were location, age, gender, tribe, siblings, birth order, class, student type, family structure, living condition, and educational status participants’ parents. For the purpose of this study, GAD-7 score ≥ 10 was set as cut-off points for diagnosis of anxiety. Chi-square test of association was done, using significance level of 5%. In order to test the independent association between the outcome variables and the covariates like age-category, birth order, family structure and ACE score, binary logistic regression was used. The magnitude of the associations between each variable and anxiety disorders, while holding other variables constant was quantified using odd ratios (ORs) at 95% confidence interval. Significance was computed at p < 0.05.

## Results

The socio-demographic profiles of the participants revealed that out of the 1187 people studied, 42% were from the rural setting, while the rest were from the urban. The mean age was 15 ± 2 years. The males were 47.5% while females constitute 52.5% of the studied population. Most of the participants were Igbo and Christians. (Table 1)

**Table 1:** Socio-demographic characteristics of the participants

		Frequency (N = 1187)	Percentage (%)
URBAN/RURAL SETTING	Rural	498	42
	Urban	689	58
AGE GROUP (YEARS)	Early Adolescence (10–13)	337	28.4
	Middle Adolescence (14–16)	628	52.9
	Late Adolescence (17–19)	222	18.7
GENDER	Male	564	47.5
	Female	623	52.5
TRIBE	Igbo	1126	94.9
	Yoruba	7	0.6
	Hausa	2	0.2
	Igala	43	3.6
	Others (e.g. Tiv, Efik)	9	0.8
RELIGION	Christianity	1179	99.3
	Islam	3	0.3
	Traditional	3	0.3
	Others*	2	0.2
SENATORIAL ZONES	Anambra North	364	30.7
	Anambra Central	373	31.4
	Anambra South	450	37.9

Mean age of participants = 14.75 years (± 1.885 years) * Others = Judaism, Hinduism, etc.

All the classes were represented with the least proportion (3.8%) coming from the examination class, SSS3. Majority of the respondents (95.6%) were day students; and about 6% of the participants came from a divorced or separated family. (Table 2)

**Table 2:** Distribution of the participants by class, student type and family structure

	Variable	Frequency (N = 1187)	Percentage (%)
Participants’ Class of study	JSS 1	226	19.0
	JSS 2	227	19.1
	JSS 3	221	18.6
	SSS 1	217	18.3
	SSS 2	251	21.1
	SSS 3	45	3.8
Student Type based on residence	Day Student	1135	95.6
	Boarder	52	4.4
Family Structure	Both parents live together	881	74.2
	Parents separated or divorced	68	5.7
	One or both parents dead	238	20.1
SIBLINGS	No Siblings	24	2.0
	1–3 Siblings	348	29.3
	4–6 Siblings	678	57.1
	**7** or more Siblings	136	11.5
BIRTH ORDER	1st	287	24.2
	2nd	218	18.4
	3rd	194	16.3
	4th	201	16.9
	5th and above	287	24.2

Parents of the participants (fathers and mothers) who attended secondary and tertiary education were more in the urban area compared to the rural areas, while those who had no formal education or stopped at primary school level were more in the rural areas. (Table 3**)**

**Table 3:** Distribution of educational status of the participants’ parents by location

Variable	Rural (n = 497)	Urban (n = 683)	Total
FATHER			
No Formal Education	42 (8.5%)	26 (3.8%)	68 (5.8%)
Primary Education	205 (41.2%)	148 (21.7%)	353 (29.9%)
Secondary Education	203 (40.8%)	347 (50.8%)	550 (46.6%)
Tertiary Education	47 (9.5%)	162 (23.7%)	209 (17.7%)
MOTHER			
No Formal Education	58 (11.7%)	22 (3.2%)	80 (6.8%)
Primary Education	183 (36.8%)	96 (14.0%)	279 (23.6%)
Secondary Education	207 (41.6%)	375 (54.8%)	582 (49.3%)
Tertiary Education	49 (9.9%)	191 (27.9%)	240 (20.3%)

**Fig. 1:**
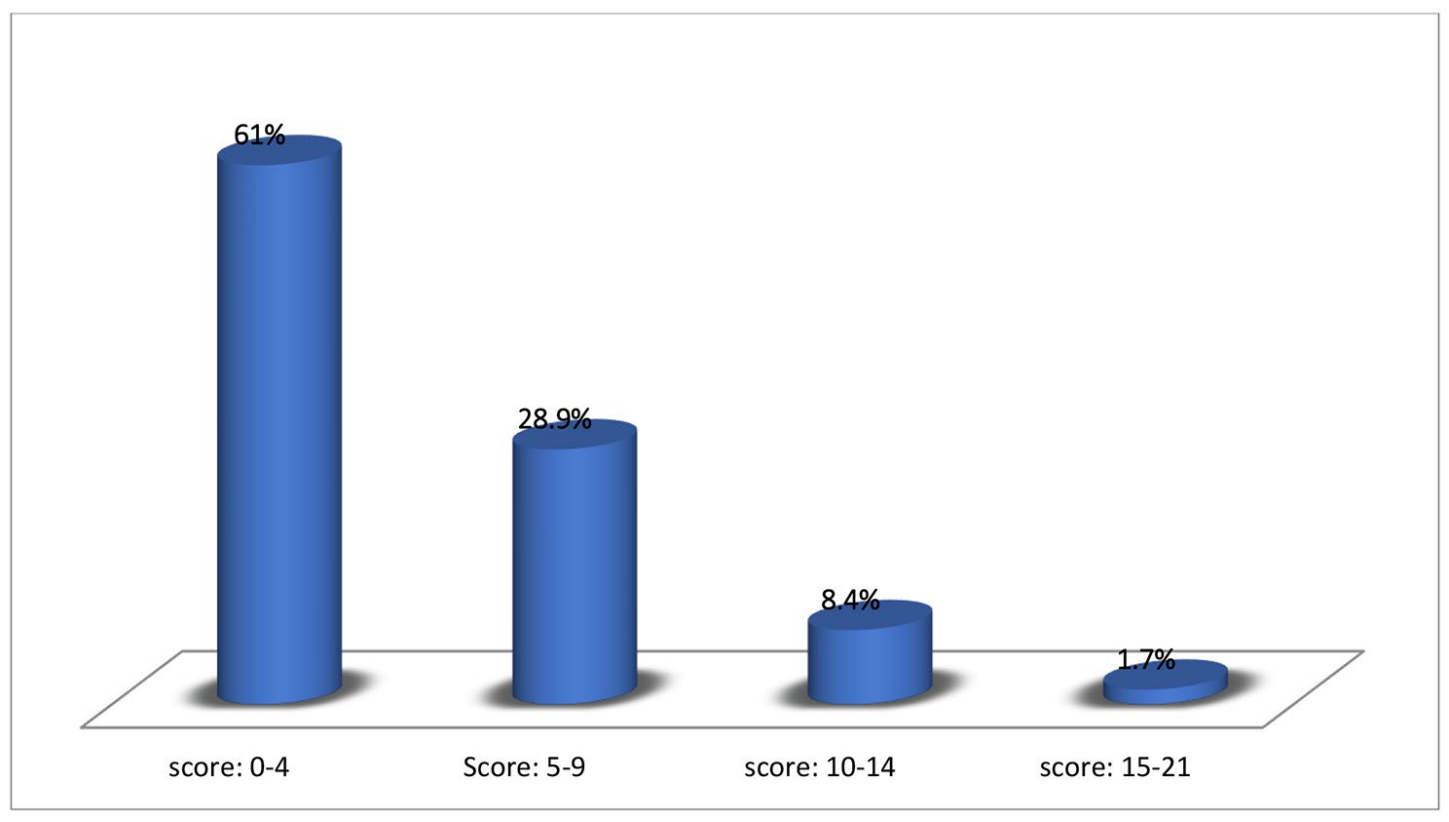
Distribution of GAD-7 scores

Out of the 1187 participants, the distribution of the scores of the GAD-7 was as follows: 724 (61%), 343 (28.9%), 100(8.4%) and 20 (1.7%) for the GAD-7 scores: 0–4, 5–9, 10–14, and 15–21 respectively. (Fig. 1)

**Fig. 2:**
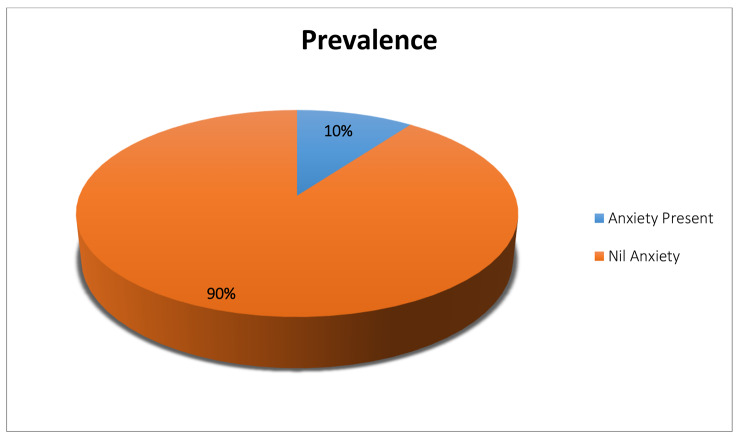
Prevalence of generalized anxiety disorder in the participants

One hundred and twenty of the participants out of the 1187 (10.1%) were found to be with generalized anxiety disorders using GAD-7 as screening tool. (Fig. 2)

The bi-variate comparison of the prevalence of probable anxiety disorders revealed that urban participants had higher prevalence compared to the rural counterparts (11% vs. 8.8%), while females had higher prevalence compared to the males in the ratio of 3:2 (or 12% vs. 8%). There was graded pattern in the prevalence of probable anxiety disorders among the 3 age groups of adolescents, with late adolescents having the highest prevalence of 14.4%. Anambra Central senatorial zone had the least prevalence of 8.6%. **(**Table 4**)**

**Table 4:** Prevalence of generalized anxiety disorders among the participants by location, gender, age group and senatorial zones

Variables		ANXIETY		
		**NO**	**YES**	***Total***
Location of participants	RURAL	454 (91.2%)	44 (8.8%)	498 (100%)
	URBAN	613 (89.0%)	76 (11.0%)	689 (100%)
Gender	MALE	519 (92.0%)	45 (8.0%)	564 (100%)
	FEMALE	548 (88.0%)	75 (12.0%)	623 (100%)
Age Group	Early Adolescence	314 (93.2%)	23 (6.8%)	337 (100%)
	Middle Adolescence	563 (89.6%)	65 (10.4%)	628 (100%)
	Late Adolescence	190 (85.6%)	32 (14.4%)	222 (100%)
Senatorial Zones	Anambra North	326 (89.6%)	38 (10.4%)	364 (100%)
	Anambra Central	341 (91.4%)	32 (8.6%)	373 (100%)
	Anambra South	400 (88.9%)	50 (11.1%)	450 (100%)

The prevalence of generalized probable anxiety disorders among the females was higher than the males even when compared based on rural and urban setting. (Table 5)

**Table 5:** Urban/rural comparison of prevalence of generalized anxiety disorders by gender

		ANXIETY			
Variables		**NO**	**YES**	***Total***	**p-value**
RURAL	Male	226 (94.2%)	14 (5.8%)	240 (100%)	0.023
	Female	228 (88.4%)	30 (11.6%)	258 (100%)	
	Total	454 (91.2%)	44 (8.8%)	498 (100%)	
URBAN	Male	293 (90.4%)	31 (9.6%)	324 (100%)	0.248
	Female	320 (87.7%)	45 (12.3%)	365 (100%)	
	Total	613 (89.0%)	76 (11.0%)	698 (100%)	

When all other variables are held constant, urban participants were found to bave 50% higher chance of being identified with symptoms of anxiety disorders compared to their rural participants (OR = 1.500, C.I.:1.002–2.246, p = 0.049). The females also had 77% significant higher chance odds of being identified with symptoms of anxiety compared to the males. Late adolescence on the other hand, were almost 3 times more likely to be identified with anxiety compared to the early adolescence. (OR = 2.75, C.I.:1.52–4.96, p = 0.001). Also, participants from divorced or separated families were about 2 times more likely to be identified with anxiety compared to those from stable family (both parents alive and living together. (Table 6)

**Table 6:** Regression of anxiety disorder on location, gender, age group, and family structure

	Slope (B)	OR (95% C.I of B)*	p-value
Location:			
Rural	-	1.000 (Reference)	
Urban	0.405	1.500 (1.002–2.246)	**0.049**
Gender:			
Male	-	1.000 (Reference)	
Female	0.571	1.770 (1.187–2.641)	**0.005**
Age Group:			
Early Adolescence	-	1.000 (Reference)	
Middle Adolescence	0.485	1.624 (0.983–2.685)	0.058
Late Adolescence	1.010	2.745 (1.519–4.960)	**0.001**
Family Structure:			
Both parents live together	-	1.000 (Reference)	
Parents separated/divorced	0.865	2.375 (1.226–4.600)	**0.010**
One or both parents dead.	0.119	1.127 (0.699–1.815)	0.624

* OR = Odds Ratio, C.I. = confidence interval

The study showed that anxiety affect how adolescents view their general health. Among the participants who gave unfavourable self-rating of their health (very poor and poor), higher proportion were identified with symptoms of anxiety (0.8% + 6.7% = 7.5%) compared to 2.7% (i.e. 0.5% + 2.2%) who had no symptoms anxiety. The proportion with favourable self-rating was higher among those without anxiety (97.3%) compared to those with anxiety (92.5%). (Table 7)

**Table 7:** Association between anxiety disorder and overall self-rating of health by the participants

Variable	ANXIETY			
	**NO (%)**	**YES (%)**	**Total (%)**	**p-value**
Very poor	5 (0.5)	1 (0.8)	6 (0.5)	< 0.001
Poor	23 (2.2)	8 (6.7)	31 (2.6)	
Good	126 (11.8)	25 (20.8)	151 (12.7)	
Very good	153 (14.3)	23 (19.2)	176 (14.8)	
Excellent	760 (71.2)	63 (52.5)	823 (69.3)	
Total	1067 (100)	120 (100)	1187 (100)	

The self-rating of ‘very poor’ and ‘poor’ constitute the ***unfavourable rating***, while the self-rating of ‘good’, ‘very good’ and ‘excellent’ constitute ***favourable rating***

## Disscusion

The overall prevalence of symptoms of generalized anxiety disorders among the in-school adolescents in Anambra State was **10.1%**. In other words, about one in ten adolescents in Anambra State will likely be identified with probable anxiety disorders. This proportion of the adolescents is significant and hence calls for attention. This study did not cover ascertaining whether preventive and promotive mental services are adequately available to cater for adolescents that may have the need. This will be for further studies. This prevalence of 10.1% is however lower compared to a prevalence of 15% reported for anxiety disorders among Nigerian secondary school adolescents from the South-West region of the country [19]. The observed disparity could be accounted for by the difference in the study design, age of study population and location. The study in South-Western Nigeria was a 12-month prevalence of DSM-IV anxiety disorders, while the present study was point prevalence [19]. The one year prevalence study is expected to be higher than the point prevalence, considering the length of time of study. Moreover, the age studied were only senior secondary adolescents aged 13–18 years (i.e. middle and late adolescence) from the semi-urban setting;[19] while the present study recruited participants aged 10–19 years, from the rural and urban areas covering both early-, middle and late adolescence. Anxiety disorders have been noted to be higher among middle and late adolescence compared to early adolescence. Hence, studying only adolescents in the middle and late stage by Adewuya and colleagues,[19] could have contributed to the higher prevalence rate. This prevalence of 10.1% in this study was based on GAD-7 cut-off point of ≥ 10. It is note –worthy that had the cut-off point being ≥ 5, the prevalence would have being higher with likelihood of more false positives, hence this studied adopted the internationally accepted cut-off point of ≥ 10 [22].

In the present study, the prevalence of anxiety among urban participants was 11% and that of the rural, 8.8%. A previous study had reported lower prevalence of anxiety for rural compared to urban dwellers [24]. The educational status of the participants’ parents in the urban area was significantly higher than that of the rural participants. One would have expected the urban participants to have a lower prevalence of anxiety disorders, but on the contrary, urban area had a higher prevalence. This points to the fact that other factors such as family disruption, media influence and social concerns could have contributed significantly to the chances of being identified with anxiety disorders [25]. This was seen also after stratification of the gender by location. The prevalence of anxiety among the males in the urban area was significantly higher than those in the rural area (9.6% against 5.8%). Similarly, the females in the urban area had higher prevalence of 12.3% compared to their rural counterparts with 11.6%.

While the prevalence of symptoms of anxiety for all participants from the rural area in the present study was 8.8%, a similar study in rural North-Eastern Uganda among children and adolescents aged 3–19 years reported a prevalence of 26.6% [17]. Most of the participants in the Ugandan study were exposed to emotional stress and trauma of war. These emotional stress and war trauma increases the likelihood of being identified with anxiety disorders. This could have accounted for the disparity with the present study.

The higher prevalence of probable anxiety disorders among females in the present study (12% against 8%) is consistent with previous studies [17, 24, 26, 27]. The female prevalence of anxiety was significantly higher than that of the males in this study even when stratified into rural and urban settings (11.6% versus 5.8% for the rural and 12.3% versus 9.6% for the urban). On the basis of age group, the prevalence of anxiety showed a ‘stepwise’ pattern of 6.8%, 10.4% and 14.4% for early-, middle- and late adolescent stage respectively. The late stage of adolescence with the highest age prevalence has been recognized as the stage with the highest influence of peers with the attendant psychological stress [28]. This psychological stress predispose to anxiety disorders. More attention need be given to adolescents especially at this critical point (late adolescence) before entering into adulthood.

Stability of the family was one of the significant factors associated with likelihood of being identified with anxiety. Adolescents with divorced (or separated) parents were seen to have more than twice the chance of having anxiety compared to those with stable family. Parents and the general public need to understand the role family stability plays in development of anxiety and possibly other mental disorders among the adolescents. The study showed that the self-image of the individual adolescents seems to be affected by the anxiety disorders as those with anxiety were more in having unfavourable self-rating of one’s health compared to those without anxiety, and vice versa. The impact of this negative self-perception of one’s health and other consequences of the identified anxiety disorders among in-school adolescents need further study.

## Conclusion

The prevalence of symptoms of anxiety disorders were found in 10% of the participants. The females were found to have a higher propensity to exhibit anxiety disorder than the males. Also reveals in this study is the fact that anxiety status affects how adolescents view their general health. These outcomes have shown that adolescents with probable anxiety disorders were prevalent in Anambra State, hence there is need to institute intervention programmes especially in the urban areas, late stage adolescence and among females, while encouraging stability in families. Interestingly, those who show signs of probable anxiety disorders were referred to specialist clinical psychologists or psychiatrists for a follow-up.

### Strengths and limitations of the study

Some of the strengths of this study include studying both rural and urban populations, whereas many of the previous studies investigated urban areas or rural areas alone. Secondly, the three senatorial zones of Anambra State were involved ensuring adequate representation of the State. Thirdly, the study had a fairly large sample size (1187 participants) from 19 schools, cutting across single sex schools and mixed schools. Lastly, standardized valid tool GAD-7, was used as instrument of study. The findings of this study may not be generalized for all adolescents in Anambra State, since only in-school adolescents participated in the study. Secondly, due to the cross-sectional and descriptive nature of the study design, temporal association cannot be established, and as well direction of causality cannot be inferred. Also, there was difficulty assessing most parents or guardians to give the informed permission. The study involved students from both day- and boarding school. In such cases, the Principal of the school or the designated authority acted as surrogate parents (loco parentis). Not having a confirmatory diagnosis, limits the research from comparing with other studies that may have had a formal diagnostic evaluation that confirmed the presence of anxiety disorder.

## Data Availability

The data is with the corresponding author and will be made available at a reasonable request.

## Abbreviations

JSS: Junior Secondary School
SSS: Senior Secondary School
GAD: Generalized anxiety disorder
SPSS: Statistical Package for the Social Sciences
IBM: International Business Machine
MASC: Multidimensional Anxiety Scale for Children
DSM: Diagnostic and Statistical Manual
OCD: Obsessive-compulsive disorder
PTSD: Post-traumatic stress disorder
LGA: Local Government Area
USA: United State of America
